# *Leishmania killicki* Imported from Tunisian Desert

**DOI:** 10.3201/eid1511.090148

**Published:** 2009-11

**Authors:** Danièle Maubon, Céline Thurot-Guillou, Christophe Ravel, Marie-Thérèse Leccia, Hervé Pelloux

**Affiliations:** Centre Hospitalier Universitaire de Grenoble, Grenoble, France (D. Maubon, C. Thurot-Guillou, M.T. Leccia, H. Pelloux); Centre National de Référence des *Leishmania* (C. Ravel); Université Montpellier 1, Montpellier, France (C. Ravel)

**Keywords:** Leishmaniasis, Leishmania killicki, emerging species, parasites, zoonoses, letter

**To the Editor:** In North Africa, cutaneous leishmaniasis (CL) is a widespread zoonosis transmitted by sandflies. In Tunisia, 3 *Leishmania* species are responsible for CL: *L. major, L. infantum*, and *L. killicki. L. major* causes 2,000–4,000 zoonotic CL infections each year. *L. infantum*, the usual agent of visceral leishmaniasis, may be implicated in sporadic CL in northern Tunisia (dermotropic strains coexist with viscerotropic strains of *L. infantum* in these areas). *L. killicki* was first described in the desert region of Tataouine, Tunisia, in 1986 (*1*) and is the agent of chronic CL. We report a case of chronic CL caused by *L. killicki,* imported to Europe by a woman who had traveled to Tunisia.

A 76-year-old woman, with no relevant medical history, sought treatment from a dermatologist in Grenoble, France, for a cutaneous lesion on her right arm. This lesion had appeared 2 months after she returned from a July 2007 trip to Tunisia, where she spent 2 weeks in the desert riding camels and sleeping under a tent. The cutaneous lesion was isolated, round, 10 mm in diameter, ulcerative, surrounded by inflammation, and painless; no lymphadenopathy was found. The patient had no lesions on her mucous membranes and no concomitant general signs or symptoms. Given the absence of substantial signs or symptoms, the patient had paid no particular attention to this lesion until it became secondarily infected with bacteria. The secondary infection resolved after treatment with antimicrobial drugs, but the lesion persisted and a diagnosis of CL, presumably caused by *L. major*, was suggested.

Histologic investigation of a skin scraping showed amastigotes of *Leishmania* spp., but no further identification was done at that time. No treatment was given because the lesion was isolated and on the arm and because *L. major* lesions frequently heal spontaneously. After 2 months, the lesion had not healed, and *Leishmania* amastigotes were still found in scrapings. After 8 months, the lesion became inflamed, and a skin scraping sample was sent to the National Reference Center of *Leishmania* in Montpellier, France. DNA was extracted, and *L. killicki* was identified by genotyping. Various therapeutic options were considered, but no clear treatment recommendations were found. Parenteral therapy (pentavalent antimonials, pentamidine isethionate, or amphotericin B) was not used because of adverse effects of these drugs. Miltefosine, used for visceral leishmaniasis (*2*), was an option because it can be taken orally and is well tolerated: however, its effectiveness for CL has mostly been evaluated for South American forms of CL, and its effectiveness for other forms is still not clear (*3*). Given the patient’s older age and the fact that lesion was isolated, intralesional treatment with meglumine antimoniate was initiated. After 9 weekly injections, the lesion disappeared (10 months after onset).

CL caused by *L. killicki* is called chronic CL because lesions persist for years, as opposed to CL caused by *L. major*, for which lesions usually resolve without treatment after a few months. *L. killicki* was first considered to appear sporadically in limited areas, but a recent study found new foci in southern Tunisia (partly overlapping an *L. major–*endemic area) (*4*,*5*) and in neighboring countries such as Algeria (*6*) and Libya (*7*). Depending on the identification method used (multilocus enzyme electrophoresis or multilocus microsatellite typing), *L. killicki* is considered to be either a separate species or a variety of *L. tropica* (*8*,*9*). Although fewer *L. killicki* cases have been reported (<20 cases since 1986), *L. killicki* infections differ from *L. tropica* infections because transmission seems strictly zoonotic (versus mostly anthroponotic for *L. tropica*) and because the clinical signs seem to be restricted to a chronic cutaneous lesion resistant to standard treatment.

The case reported here highlights the effect of ecotourism on imported diseases; journeys that were previously considered adventurous (i.e., physically challenging) are now easily accessible to anyone, thanks to the operation of well-organized tours. When treating travelers, clinicians must be aware of the specific epidemiology of disease agents in the regions visited. This case also shows the difficulties encountered when selecting treatment for leishmaniasis because it is still considered a neglected tropical disease, and thus the development of effective and nontoxic drug treatments has not been a priority. Lastly, this case shows how travelers can potentially spread rare diseases. Reservoirs and vectors can also be imported to other regions as a result of urbanization, climate change, and exportations. These factors lead to changes in environmental conditions favorable to spread of anthroponotic *Leishmania* spp. (urbanization) or to the establishment of tropical and/or subtropical vector species (global warming), and *Leishmania* strains can be exported through dogs. Specific species of *Leishmania* are no longer circumscribed to particular regions. In Europe, controlling this vector-borne disease seems essential because leishmaiasis is already endemic to southern Europe and new species may be introduced (*10*).

CL caused by *L. killicki* will probably soon become more common among persons who travel throughout North Africa. Because of the chronic evolution of CL lesions, clinicians should characterize the *Leishmania* strain and, if necessary, adapt their patient care to the strain.
